# Microwave-Assisted Extraction and HPLC-UV-CD Determination of (S)-usnic Acid in *Cladonia foliacea*

**DOI:** 10.3390/molecules26020455

**Published:** 2021-01-16

**Authors:** Valeria Cavalloro, Giorgio Marrubini, Rita Stabile, Daniela Rossi, Pasquale Linciano, Gabriele Gheza, Silvia Assini, Emanuela Martino, Simona Collina

**Affiliations:** 1 Department of Earth and Environmental Sciences, University of Pavia, 27100 Pavia, Italy; valeria.cavalloro01@universitadipavia.it (V.C.); silviapaola.assini@unipv.it (S.A.); 2 Department of Drug Sciences, University of Pavia, 27100 Pavia, Italy; giorgio.marrubini@unipv.it (G.M.); rita.stabile01@universitadipavia.it (R.S.); daniela.rossi@unipv.it (D.R.); simona.collina@unipv.it (S.C.); 3 Department of Biological Geological and Environmental Sciences University of Bologna, 40126 Bologna, Italy; gheza.gabriele@gmail.com

**Keywords:** *Cladonia foliacea*, usnic acid, chiroptical properties, absolute configuration, HPLC-UV/PAD-CD, DoE, MAE, lichens

## Abstract

During the years, many usnic acid (UA) conjugates have been synthesized to obtain potent endowed with biological properties. Since (*S*)-UA is less abundant in nature than (*R*)-enantiomer, it is difficult to source, thus precluding a deeper investigation. Among the lichens producing UA, *Cladonia foliacea* is a valuable (S)-UA source. In the present work, we report on a rapid HPLC-UV/PAD-CD protocol suitable for the analysis and the identification of the main secondary metabolites present in *C. foliacea* extract. Best results were achieved using XBridge Phenyl column and acetonitrile and water, which were both added with formic acid as mobile phase in gradient elution. By combining analytical, spectroscopical, and chiroptical analysis, the most abundant analyte was unambiguously identified as (*S*)-UA. Accordingly, a versatile microwave-assisted extractive (MAE) protocol, assisted by a design of experiment (DoE), to quantitatively recover (*S*)-UA was set up. The best result in terms of UA extraction yield was obtained using ethanol and heating at 80 °C under microwave irradiation for 5 min. Starting from 100 g of dried *C. foliacea*, 420 mg of (*S*)-UA were achieved. Thus, our extraction method resulted in a suitable protocol to produce (*S*)-UA from *C. foliacea* for biological and pharmaceutical investigation or commercial purposes.

## 1. Introduction

**Figure 1:**
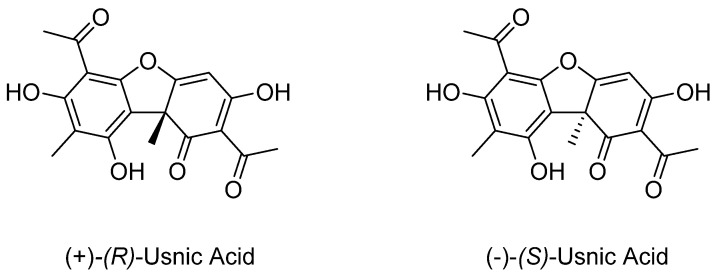
Chemical structure of usnic acid (UA) enantiomers.

Metabolites produced by lichens are still poorly investigated, although these organisms are commonly used in folk medicine to treat pathological conditions [1,2,3,4,5,6,7,8,9]. Among the most investigated metabolites, usnic acid (UA) deserved attention due to its intrinsic properties and its potential use as chiral synthon. UA is a dibenzofuran derivative, and it is characterized by the presence of a stereogenic center (Figure 1). Both enantiomers occur in nature, depending on the producing organism. The (*R*)-configured enantiomer of UA is the most abundant in nature, and *Cladonia arbuscula*, *C. mitis*, *Ramalina boninensis*, *R. pacifica*, *R. roesleri*, *R. farinacea*, *Usnea diffracta*, *U. longissima*, *U. hirta*, *U. steineri*, *Flavoparmelia caperata*, and *Xanthoparmelia chlorochroa* were identified as valuable source for its extraction. Conversely, (*S*)-UA was isolated as an exclusive enantiomer in few species of *Cladonia* (*C. uncialis* and *C. foliacea*) and *Alectoria* (*A. lata* and *A. ochroleuca*) [10]. In another few species of lichens, UA was detected as a mixture of (*R*)- and (*S*)-isomers in different ratios [10].

**Figure 2:**
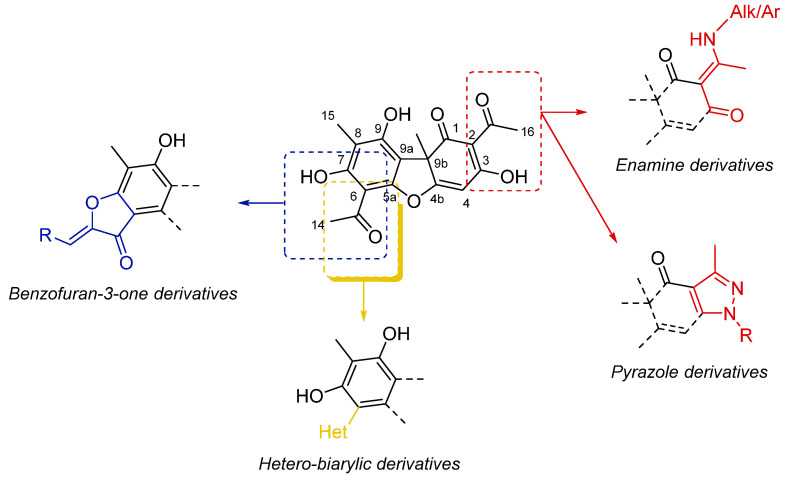
Chemical modifications of the main core scaffold of UA apported in the derivatives described in the literature [13,15,16,17,18,19,20,21,22,23,24,25].

UA has demonstrated interesting bioactivity from a biological standpoint, with antimicrobial, cytotoxic, or anti-inflammatory properties [10]. Moreover, UA was exploited as a useful intermediate to obtain potent analogs with improved biological profiles [11,12,13,14]. The chemical modifications introduced in the main scaffold of UA are resumed in Figure 2 [13,15,16,17,18,19,20,21,22,23,24,25]. All these chemical modifications preserve the stereochemistry of the UA used as starting material.

However, most of the published data concern (*R*)-UA, whereas only a few studies regarding (*S)*-UA are described. Moreover, only a small number of reports refer to (R)- and (S)-UA activity when examined in comparison. The discrepancy about the investigations around the (*R*)- and (*S*)-UA results from the different accessibility and availability of the two enantiomers. Indeed, (*R*)-UA is commercially available due to the high abundance of accessible natural sources. In contrast, (*S*)-UA is less naturally abundant. It is not available from the leading chemical suppliers; otherwise, it is sold at a retail cost 30 times higher than the (*R*)-enantiomer. Therefore, the prior extraction of (S)-UA was required before proceeding with the biological or pharmacological investigation or the further chemical modification, thus hugely limiting the use and investigation of the (*S*)-UA so far.

**Figure 3:**
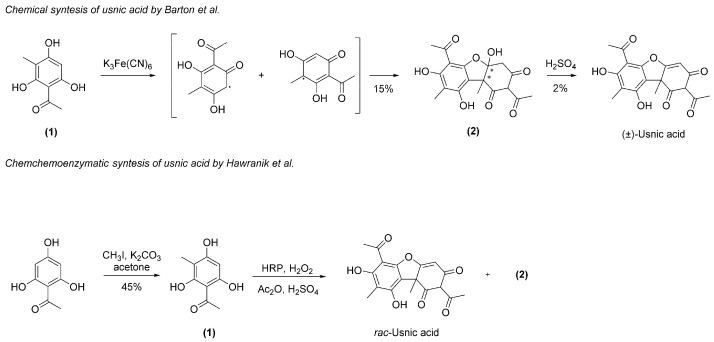
Chemical and chemoenzymatic synthesis of UA.

As an alternative to the extractive procedure for the production of UA, only two synthetic strategies have been reported (Figure 3) [26,27].

These processes suffer from several drawbacks such as the availability of the starting materials, the employment of hazardous reactive or of hard to handle enzymes, the cumbersome purification procedures, and the difficulty to control the stereochemistry. Indeed, following these two procedures, UA was obtained exclusively as a racemic mixture and in poor yield (overall yield 2%) or in mixture with the hydrated derivative **(2)**. Thus, the extraction of enantiomeric (*R*)- or (*S*)-UA from lichens remains the preferred procedure. To the best of our knowledge, the extraction protocols for UA reported in the literature mainly exploit maceration, Soxhlet, or supercritical CO_2_ extraction, using diverse solvents such as water, methanol, ethanol, acetone, or benzene and with an extraction time ranging between 1 and 6 h. Several analytical procedures aimed at identifying and quantifying UA in the dry extract or biological fluids, including High-Performance Liquid Chromatography (HPLC)/HPLC coupled with MS or UV detector, High-Performance Thin Layer Chromatography (HPTLC) and Thin Layer Chromatography (TLC), were developed [28,29,30,31,32,33,34,35,36,37].

**Figure 4:**
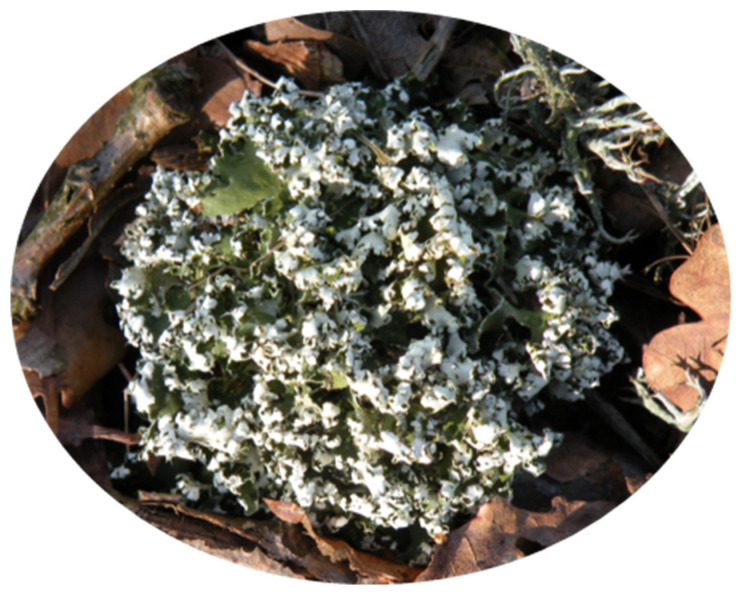
*Cladonia foliacea*, photo snapped.

Starting from these considerations, herein, we focused on *Cladonia foliacea* as a valuable source of this metabolite [38,39,40]. *C. foliacea* (*Cladoniaceae*, *Lecanorales*, lichenized *Ascomycota*) is a soil-dwelling lichen with a leafy appearance, being its thallus composed of squamules organized in pads (Figure 4). *C. foliacea* has been already reported as one of the few lichen species able to exclusively produce the (S)-enantiomer of UA. Moreover, *C. foliacea* is widespread in Eurasia, occurring only in open dry habitats on oligotrophic soils in lowlands and hills, mostly under temperate climate. *C. foliacea* spontaneously grows in inland sand dunes in the western Po Plain (Lombardy region, Nord Italy). Considering this, studies about this interesting species can lead not only to the valorization of our biodiversity but also to the conservation and protection of the open dry habitats, which typically host this and other *Cladonia* species [41,42,43].

In the present work, we report on a rapid HPLC-UV/PAD-CD analytical methodology suitable for the analysis and the identification of the main secondary metabolites present in *C. foliacea* crude extract. By combining spectroscopical and chiroptical analysis, the main secondary metabolites present in the crude extract have been identified, with (*S*)-UA resulting in the most abundant. To exhaustively recover (*S*)-UA from *C. foliacea*, A versatile microwave-assisted extractive (MAE) protocol, developed by a design of experiment (DoE), was then set up.

## 2. Results

### 2.1. Isolation and Characterization of the Main Metabolites Present in C. foliacea Extract

Thalli of *C. foliacea* were harvested from a lowland dry grassland located at *Bosco della Ghisolfa* (Province of Pavia, Northern Italy). They were used to prepare a pilot extract by an in-house consolidated microwave-assisted extraction (MAE) protocol [44,45,46,47,48,49,50,51]. The natural matrix was subjected to microwave irradiation, using acetone as extractive solvent [52].

The solvent was evaporated. The obtained crude was used to set up a rapid and economic HPLC-UV/PAD-CD protocol that was suitable for the analysis of the content of the lichen extract and for gaining information on the stereochemical properties of the analytes present within. Two different stationary phases with complementary selectivity were investigated: a classical C-18 reversed-phase and an XBridge Phenyl stationary phase. The latter contains bonded aromatic ligands, and therefore, it is useful for analyzing polyaromatic compounds. The best results in terms of the time of analysis, peak shape, and peak resolution were achieved using the XBridge Phenyl (5 μm, 4.6 × 150 mm) column. Acetonitrile (A) and water (B), both added with formic acid (0.1%, *v*/*v*), were used as mobile phase. A gradient elution was set up: from 75% to 100% of A in 7 min, followed by a 100% isocratic of A for 1 min.

**Figure 5:**
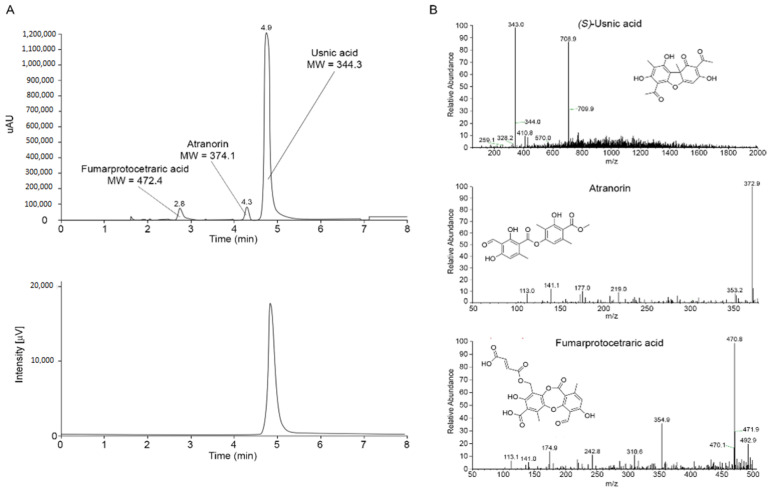
(**A**) HPLC-PAD profile of the crude acetone extract of *C. foliacea* recorded at λ = 254 nm (above) and the corresponding HPLC-CD profile recorded at λ = 270 nm (below); (**B**) ESI-MS spectra of the peaks at 4.9, 4.3, and 2.8 min and ascribable to UA, atranorin, and fumarprotocetraric acid, respectively.

The chromatographic HPLC-UV/PAD-CD profile of the crude extract is reported in Figure 5A. Three prominent and fully resolved peaks were detected in the UV trace at 2.8, 4.3, and 4.9 min (Figure 5A, above). To investigate the chiroptical properties of the analytes present in the crude extract, the in-line circular dichroism of the eluate was registered at 270 nm. The CD traces of the crude extract showed that the two peaks at 2.8 and 4.3 min were optically inactive, whereas the peak at 4.9 min registered a positive Cotton effect (Figure 5A, below).

The chemical identification of the three detected secondary metabolites was investigated, combining analytical and spectroscopical approaches [53]. Therefore, the crude extract was subjected to HPLC-ESI-MS to determine the m/z ratio of the three metabolites, which resulted in 473, 375, and 344 m/z for the compounds eluting at 2.8, 4.3, and 4.9 min, respectively. The ESI mass spectra of the three analytes are reported in Figure 5B. By querying online spectral databases for natural product identification, the molecular mass and mass spectra of the three analytes detected in the crude extract of *C. foliacea* were compatible with those of fumarprotocetraric acid (t_R_ = 2.8 min, ESI-MS [M-H]^−^ calcd for C_22_H_15_O_12_^−^: 471.1; found 471.8; MW = 472.4), atranorin (t_R_ = 4.3 min, ESI-MS [M-H]^−^ calcd for C_19_H_17_O_8_^−^: 373.1; found 372.9; MW = 374.1), and UA (t_R_ = 4.9 min, ESI-MS [M-H]^−^ calcd for C_18_H_15_O_7_^−^: 343.1; found 343.0; MW = 344.3). These three proposed secondary metabolites are highly plausible, since fumarprotocetraric acid, atranorin, and in particular UA have been already detected in *C. foliacea*. Moreover, the proposed structures are in accordance with the results observed in the HPLC-CD analysis: fumarprotocetraric acid and atranorin are achiral compounds, whereas UA, unless in a racemic mixture, may exert a Cotton effect in CD spectra.

The three analytes were purified from the crude extract to confirm the identity of the main three secondary metabolites and the stereochemistry of the putative UA. The crude was precipitated first over dichloromethane, and the precipitated was collected by filtration. By HPLC-UV/PAD-CD analysis, it resulted in the analyte eluting at 4.3 min and therefore was ascribable to atranorin. Later, the organic phase was concentrated, and the residue was partitioned between water and hexane. After collection and evaporation of the organic phase, the secondary crude obtained was further purified over silica gel to achieve two analytic samples of the compound eluting at 2.8 min (putative fumarprotocetraric acid) and 4.9 min (putative UA). The purity of the three analytes was assessed by HPLC analysis, and the chemical identification was achieved by mono- and bidimensional NMR spectroscopy. The comparison of the NMR spectra of compounds eluted at 2.8 min (*m*/*z* = 473) and 4.3 min (*m*/*z* = 375) with the data reported in the literature confirmed them as fumarprotocetraric acid and atranorin, respectively (Figure S1-2), [54,55].

**Figure 6:**
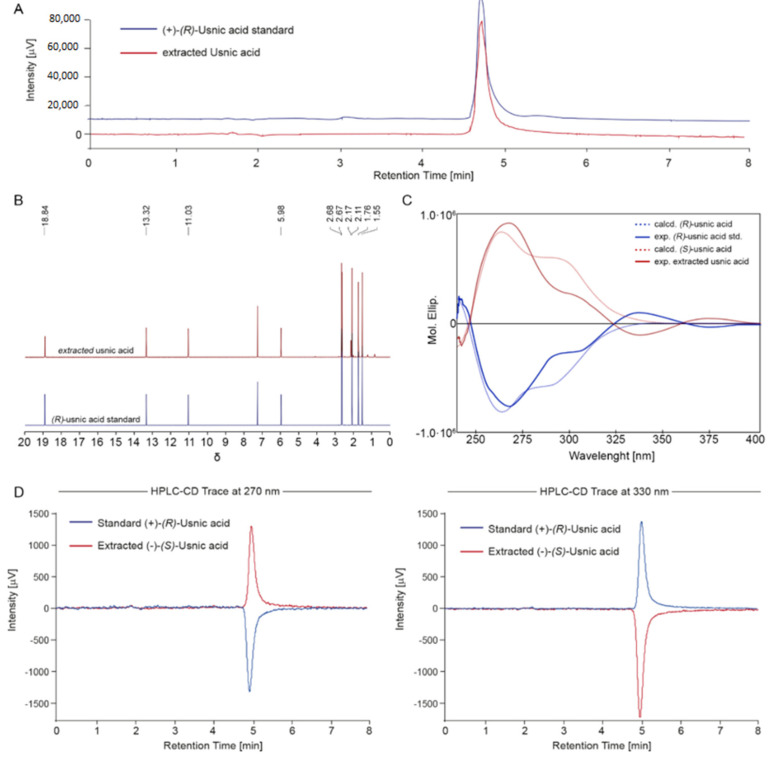
Superimposition of the (**A**) HPLC-UV chromatogram, (**B**) ^1^H NMR spectra, (**C**) electronic circular dichroism (ECD) spectra in chloroform and (**D**) HPLC-CD traces acquired at 270 and 330 nm of extracted (S)-UA (in red) and (R)-UA standard.

The most abundant analyte identity, hypothesized to be UA, was instead thoroughly investigated by comparing its analytical, spectroscopical, and chiroptical properties with those of the commercially available (R)-UA, which was purchased and used as reference standard. A perfect superimposition between the analytical HPLC-UV profile (Figure 6A) and proton chemical shifts of both (R)-UA standard (blue spectra, Figure 6B) and extracted metabolite (red spectra, Figure 6B) was observed, thus confirming the identity of the most abundant analyte as UA. In addition, a full spectroscopic NMR characterization, comprising ^13^C, HSQC, and HMBC, supported this conclusion (Figure S3). Chiroptical analysis, such as electronic circular dichroism (ECD) and optical rotatory power, was performed to assign the absolute configuration of the extracted UA.

The ECD spectra of extracted UA in chloroform were acquired and compared with the ECD spectra of the standard (R)-UA. As depicted in Figure 6C, the UA from *C. foliacea* (red line) showed an ECD profile entirely specular with respect to the standard (blue line). In detail, the ECD spectra of extracted UA showed two positive Cotton effects at 270 and 300 nm and one negative Cotton effect at 330 nm. In contrast, the standard (R)-UA presented the same prominent bands but with an opposite profile. Moreover, the ECD spectra for both (S)- and (R)-UA were computationally calculated. The results were in accordance with the experimental ECD spectra, thus supporting the right assignment of the absolute configuration.

Since the ECD profile of UA shows a main Cotton effect (CE) at 250 nm with a shoulder at 325 nm and an opposite CE at 360 nm, the HPLC-CD spectra have been recorded at 275 and 320 nm. Wavelengths below and around 250 nm have been avoided due to excessive CD noise observed in the region. This noise is due to the absorbance of acetonitrile, which was added to the HPLC mobile phase. According to experimental and calculated EDC spectra, the HPLC-CD chromatograms recorded at 275 nm showed a negative CE for the standard (R)-UA and a positive CE for the extracted UA (Figure 6D, left). Conversely, at 330 nm, the HPLC-CD traces were positive for (R)-UA and negative for (S)-UA (Figure 6D, right). Taken together, these data confirm that it is essential to record the ECD spectra, or at least to calculate them, prior to setting the in-line CD wavelength, especially when the enantiomeric analyte is responsible for CEs of opposite sign, depending on the selected wavelength.

The (S)-enantiomer of UA herein isolated was used as an external standard, and an instrumental calibration via a six-point calibration curves, each replicated three times, was performed. The extracted (S)-UA showed an [α]_D_^25^ of −476° (c = 0.5%, chloroform), which is in line with the absolute [α]_D_^25^ value of the standard (R)-enantiomer reported in the datasheet of the supplier ([α]_D_^25^ of + 488°, c = 0.7%, chloroform, from Carbosynth, Staad, Switzerland). By comparison of the absolute value of the optical rotation, we can state that the optical purity of the herein extracted UA is c.a. 99% (based on the assumption that the optical purity of the standard was about 100%) and that no racemization occurred during the extraction procedure [56].

To conclude, the (S) absolute configuration can be undoubtedly assigned to the UA extracted from *C. foliacea*. The fully characterized (S)-UA and was used as standard in further experiments.

### 2.2. Microwave-Assisted Extraction (MAE) Protocol

To assess the experimental conditions suitable to extract exhaustively (S)-UA, a design of experiments (DoE) methodology was applied [57,58]. Briefly, the DoE approach is a collection of systematic statistical-based tools to study chemical processes both in the academic and industrial fields. Initially developed by Sir Ronald A. Fisher [59,60], many authors have continuously implemented it to provide the scientific community with tools that could minimize the investments in labor and time while maximizing the information obtainable by rigorous experimentation [61,62,63]. DoE is increasingly used and can be applied to several kinds of experiments, i.e., organic synthesis [64], analytical method development [65], and sample preparation [66]. Particularly, factorial designs represent an attractive approach for natural matrices extraction [48] and the systematic evaluation of plant secondary metabolism [67].

In contrast with the classic “one factor at a time” approach, DoE permits the study of the influence of multiple factors simultaneously. Specifically, two responses were studied varying three different parameters: solvent, extraction time, and temperature. Acetone, ethyl acetate, and ethanol characterized by different polarity and dielectric constant have been selected as extraction solvent, since it is well known that the microwave heating response depends on the solvent used.

We considered the total extraction yield percentage (Y_1_) and the (-)-(S)-UA % recovered (Y_2_).

The quantitative UA determination was performed by HPLC-UV/PAD using the same protocol exploited for the identification of UA in the lichen dry extract. The (S)-enantiomer of UA herein isolated was used as an external standard and six-point calibration curves, each replicated three times, were determined. The method response function was linear with a good correlation coefficient (R) of 0.9996 (y = (2.58 ± 0.04) × 10^7^x − (3 ± 2) × 10^5^). The statistical control of the method was assessed by performing three injections of external standard at the beginning of every measurement session on different days. The limits of detection and quantification were also estimated during the validation procedure using the S/N ratio. S was at least 3N in the peak region of the chromatogram for the limit of detection (LOD), whereas S > 10N for the limit of quantification (LOQ). The values resulted in being 0.0063 mg/mL and 0.0022 mg/mL, respectively, and were also experimentally confirmed. The method repeatability and accuracy were assessed by performing three injections on different days and assessing the UA recovery by a double-point standard addition method, spiking two aliquots of 0.30 g of sample with 12 mg and 9 mg of (-)-(S)-UA. Good recovery has been obtained, being the comparison between the spiked amount of UA and the amount found in the non-spiked samples as high as 99%. To sum up, the method is suitable for (S)-UA quantification in *C. foliacea* extracts. Results are reported in Table 1.

The best results in terms of UA extraction yield (Y_2_), whose quantification is the primary goal of the present study, were obtained using ethanol, applying two cycles of microwave heating of 5 min each at 80 °C (experiment number 11, Table 1).

To obtain an insight into thermal (S)-UA degradation, an additional MAE experiment (70°C, two cycles of 5 min, ethanol) was carried out, adding known amounts of (S)-UA to the natural matrix. The good recoveries obtained (about 90%) confirmed the chemical stability of UA under the experimental conditions. With the extraction method in hand, the extraction procedure was scaled up. In detail, 100 g of dried *C. foliacea* were extracted, exploiting the parameters of experiment 11, thus obtaining 420 mg of (-)-(S)-UA (yield, 0.42%, HPLC purity, 99.9%). The extracted (-)-(S)-UA was analyzed by HPLC-UV/PAD-CD, the optical rotation was measured, and the analytical data were compared with those of the commercial (R)-UA standard and the (S)-UA standard previously prepared and characterized as described above. Based on the results obtained, we can state that the applied extraction protocol does not induce racemization.

## 3. Materials and Methods

### 3.1. Lichen Material

Thalli of *C. foliacea* were harvested from a lowland dry grassland located at Bosco della Ghisolfa (Province of Pavia, Northern Italy) in Spring 2018.

Plant material was reduced to a homogenous powder by grounding it with a blade-mill (A10 IKA-Werke GmbH & Co. Staufen, Germany) just before performing the extractions.

### 3.2. Chemicals

Formic acid and deuterated solvents were purchased from Sigma-Aldrich (Milan, Italy).

HPLC-grade solvents were supplied by Honeywell (Seelze, Germany), while analytical grade solvents were supplied by PanReac (Barcelona, Spain).

Standard (R)-UA was purchased by Carbosynth (Staad, Switzerland).

### 3.3. Instruments

The evaporation procedures were performed under reduced pressure using a Heidolph Laborota 4000 instrument (Heidolph Instruments GmbH & Co., Schwabach, Germany). Analytical thin-layer chromatography (TLC) was carried out on silica gel pre-coated glass-backed plates (Fluka Kieselgel 60 F254, Merck, Darmstadt, Germany). The detection was conducted with UV light (λ = 254 nm). Flash chromatography was performed with silica gel 60 (particle size 230–400 mesh) purchased from Nova Chimica (Cinisello Balsamo, Italy). Separations were carried out at room temperature, and separations were obtained using two different columns from Waters (Waters Corporation, Milford, MA, USA), namely a Symmetry C-18, 5 μm, 150 × 3.9 mm and an XBridge Phenyl 5 μm, 4.6 × 150 mm. Analyses were carried out using the following systems. HPLC-UV/PAD-CD: a Jasco (Tokyo, Japan) system consisting of a PU-1580 pump and a MD-1510 photodiode array (PDA) detector. Chromatogram acquisitions and elaborations were performed using the ChromNAV software (Tokyo, Japan). HPLC-UV/PAD-ESI/MS: Finnigan LCQ fleet ion trap system, controlled by Xcalibur software 1.4 (Thermo Finnigan, San Jose, CA, USA). Optical rotation was recorded using a Jasco photoelectric polarimeter DIP 1000. Electronic circular dichroism (ECD) spectra were recorded on a Jasco J-1500 Circular Dichroism Spectrophotometer from 400 to 240 nm. Nuclear magnetic resonance spectra were recorded on Bruker Avance 400 spectrometers operating at 400 MHz. Chemical shifts (δ) are reported in parts per million with the solvent reference relative to tetramethylsilane (TMS) internal standard.

### 3.4. Isolation and Identification of Main Metabolites

#### 3.4.1. Isolation and Identification of Main Metabolites

A preliminary extract was prepared by applying an MAE methodology on 10 g of natural matrix using acetone as a solvent (2 min ramping, maximum pressure 120 psi, maximum potency 100 W, 80 °C) for three cycles of 5 min each. Then, the extract was filtered, and the solvent was evaporated under reduced pressure. The green syrup so obtained was suspended in dichloromethane (DCM). The suspension was filtered, and the solid residue was washed two times with DCM, thus obtaining atranorin as white powder, as confirmed by NMR analysis. Then, the combined organic phases were concentrated under reduced pressure and purified by L/L extraction (water/hexane). The aqueous phase was discarded, and the combined organic phases were concentrated under reduced pressure. After flash chromatography (diethyl ether: n-hexane (6: 4, *v*/*v*) added with formic acid (0.1%, *v*/*v*)), (−)-(S)-UA as yellow powder and 3 mg of fumarprotocetraric acid have been isolated.

Fumarprocetraric acid, 5 mg, mp 257°C, *m*/*z* 473; ^1^H-NMR in DMSO-d_6,_ δ (ppm): 10.57 (s, 1H, CO**H**), 6.82 (s, 1H, C**H**), 6.62 (s, 1H, C**H**CH), 6.62 (s, 1H, CHC**H**), 5.28 (s, 2H, C**H_2_**), 2.46 (s, 3H, C**H_3_**), 2.42 (s, 3H, C**H_3_**).

Atranorin, 2mg, mp 190°C, m/z 375; ^1^H-NMR in CDCl_3_, δ (ppm): 12.59 (s, 1H, O**H**), 12.53 (s, 1H, O**H**), 11.98 (s, 1H, O**H**), 10.40 (s, 1H, CO**H**), 6.55 (s, 1H, C**H**), 6.44 (s, 1H, C**H)**, 4.02 (s, 3H, OC**H_3_**), 2.72 (s, 3H, C**H_3_**), 2.58 (s, 3H, C**H_3_**), 2.13 (s, 3H, C**H_3_**).

(-)-S-UA, 15 mg, mp 204 °C, m/z 344, [α]_D_^25^= −476°, c 0.5% CHCl_3_, **^1^**H-NMR in CDCl_3_ δ (ppm): 18.86 (s, 1H, O**H**), 13.33 (s, 1H, O**H**), 11.05 (s, 1H, O**H**), 6.00 (s, 1H, OCC**H**), 2.70 (s, 3H, COC**H_3_**), 2.68 (s, 3H, COC**H_3_**), 2.13 (s, 3H, CCC**H_3_**), 1.78 (s, 3H, COCC**H_3_**) (see Figure S3 for complete NMR characterization).

#### 3.4.2. HPLC-UV/PAD-CD and HPLC-UV/PAD-ESI/MS Analysis

A proper high-performance liquid chromatography-electrospray-tandem mass spectrometry (RP-HPLC-UV/PAD-ESI/MS) method was set up, analyzing the crude extract, atranorin, (-)-(S)-UA and fumarprotocetraric acids isolated, as described in Section 3.4.1.

The best results in terms of time of analysis and peak resolution were obtained at 1 mL/min flow rate at room temperature using an XBridge Phenyl column (5 μm, 4.6 × 150 mm). The mobile phase consisted of water (A) and acetonitrile (B), which were both added with 0.1% (*v*/*v*) of formic acid. The eluent was applied onto the column in gradient mode from 75% to 100% B in 7 min, which was followed by an isocratic elution step for 1 min. The column was reconditioned by eluting from 100% B to 75% B in 1 min and with a final 4 min isocratic elution at the initial conditions. Mass spectra were generated in positive and negative ion mode (mass range: 50–2000 Da, capillary temperature 120 °C): ion spray voltage 3 kV, capillary voltage 10 V, aux gas flow rate 10, sheath gas flow rate 20, and tube lens voltage 75 V for positive mode and ion spray voltage 5 kV, capillary voltage −45 V, aux gas flow rate 10, sheath gas flow rate 20, and tube lens voltage −125 V for negative mode. MS/MS data were acquired in dependent scan mode (full-scan MS followed by MS/MS of the most intense ion). The identification of (S)-UA was unequivocally accomplished by comparing its HPLC retention time, UV, and MS spectra with those of the standard analyzed in the same conditions.

The same method was also applied to a HPLC-UV/PAD-CD system. Analytes were detected photometrically at 220 and 254 nm.

#### 3.4.3. Electronic Circular Dichroism

The chloroform solutions of (–)-(S)-UA (c: 1.09 × 10^−6^ M) and (+)-(R)-UA (c: 1.37 × 10^−6^ M) were analyzed in a nitrogen atmosphere with an optical pathway of 1 cm. ECD spectra were scanned at 200 nm/min with a spectral bandwidth of 1 nm and a data resolution of 1 nm. For each measurement, 10 scans were taken and averaged, considering both enantiomers. ECD spectra of the solvent in the same experimental conditions were subtracted. Data are reported in Δε versus λ (nm) from knowledge of the cell path length and solution concentration (Figure 6C).

#### 3.4.4. Computer-Assisted Conformational Analyses and ECD Calculations

Conformational analyses of (R)- and (S)-UA were performed with Merck Molecular Force Field (MMFF) by using Maestro 10.3 of the Schrödinger Suite. The lowest energy conformers of (R)- and (S)-UA were subjected to ECD calculation, which was carried out with the B3LYP/6-31G(d,p) level of time-dependent density functional theory (TDDFT). The polarizable continuum model (PCM) was used to take into account the solvent effects of chloroform. The calculated ECD spectra of (R)- and (S)-UA were the result of a weighted average of the calculated ECD spectra of each conformer based on Boltzmann distribution.

#### 3.4.5. Quantitative Determination of (−)-(S)-UA in the Extract

Quantitative determination of (−)-(S)-UA was performed using the (−)-(S)-enantiomer isolated in the preliminary part of this work as an external standard, exploiting the HPLC/UV-PAD protocol used for the UA identification in the dry extract. The calibration curve was built through six points, each replicated three times. The limits of detection (LOD) and quantification (LOQ) were estimated using the S/N ratio. S was at least 3N in the peak region of the chromatogram for the LOD, whereas S > 10N for the LOQ. The values resulted in being 0.0063 mg/mL and 0.0022, respectively. These data were also experimentally confirmed. However, the calibration curve was studied in the range of 0.75–0.1 mg/mL. The method response function was linear with a correlation coefficient (R) of 0.9996 (y = (2.58 ± 0.04)×10^7^ x −(3 ± 2)×10^5^). The statistical control of the method was assessed by performing three injections of external standard at the beginning of every measurement session on different days. The method accuracy was evaluated by assessing the UA recovery by a double-point standard addition method, spiking two aliquots of 0.30 g of sample with 12 mg and 9 mg of (−)-(S)-UA, respectively. Each experiment has been repeated in triplicate. The recovery found by comparing the spiked amount of UA to the amount found in the non-spiked samples was 99.3 ± 0.2% for samples spiked with 12 mg of UA and 99.1 ± 0.3% for samples spiked with 9 mg of UA.

#### 3.4.6. Experimental Design of the Extraction Procedure

The experimental design of the extraction procedure was aimed at the screening of the factors that influenced two responses, namely the total extraction yield (%, Y_1_) and the yield of (−)-(S)-UA extracted per unit amount of source material (mg (−)-(S)-UA/g of lichen, Y_2_).

Three process factors were considered. These parameters are all known to strongly influence the extraction procedures and are also strictly related to microwave power. The first factor was the extraction solvent. Three solvents were selected having different responses to microwave heating, namely ethyl acetate (S_1_, polarity index = 4.4, LogP 0.71, low microwave absorbance), acetone (S_2_, polarity index = 5.1, LogP −0.24, medium microwave absorbance), and ethanol (S_3_, polarity index = 5.2, LogP −0.31, high microwave absorbance).

These solvents have also been chosen because they are suitable for the scaling-up process, being considered the safest among the available.

The second factor was the time of extraction (2 × 5 – 3 × 5 number of cycles of microwaves irradiation per minute, X_2_).

The third factor was the extraction temperature (60–80°C, X_3_).

Given these factors and levels, a full factorial design, including 2^2^ × 3 = 12 experiments, was used as an experimental plan (Table 1).

A weight of 0.30 g of ground plant material was dispersed in 6 mL of solvent (acetone, ethyl acetate, or ethanol (Table 1)) under magnetic stirring and subjected to microwave heating (2 min ramping, maximum pressure 120 psi, maximum potency 100 W, at 60°C or 80°C (Table 1)) for 2 to 3 cycles of 5 min each (Table 1). Then, the samples were cooled to 40°C and filtered through a paper filter. The solvent was evaporated under reduced pressure to obtain a yellow-green oil (yield, Table 1).

### 3.5. Experimental Design Analysis and Extraction Efficiency Enhancement

The full factorial 2^2^·3 experimental design selected counted twelve experiments. One additional test experiment was performed in three independent replicates and kept apart from those used to build the model (Table 1, Exp#13). The test experiment was used to verify whether the models computed for the two responses could reliably represent the responses and assess the overall method standard deviation in the experimental domain center. This procedure represents the model’s validation and consists of comparing the results predicted by the models with the outcome of the test experiment. The models will be accepted as reliable if the predicted data agree with the test experiment results within limits of uncertainty acceptable for the method application (Table 1).

The response Y_1_, extraction yield percentage, is modeled reliably by the linear model, according to the equation:

Y_1_= 6.62 − 0.27·X_2_ − 0.13·X_3_ − 3.00·S_1_ − 3.90·S_2_ + 0.18·X_2_ X_3_.

The (−)-(S)-UA yield (Y_2_) results were modeled by the equation

Y_2_ = 12.66 − 0.57 X_2_ + 0.25·X_3_ − 0.02·S_1_ − 0.79·S_2_ + 0.00 X_2_ X_3_.

Both models predict reliably the experimental results with the predicted data, which refer to the experiment with ethanol irradiated with two cycles of 5 min each, at 70°C, i.e., in the test point having coded coordinates S_1_ = S_2_ = 0, X_2_ = −1, and X_3_ = 0. However, while the model for Y_1_ (yield%) shows a good fit (r = 0.935), the model for (−)-(S)-UA yield (Y_2_) shows limited fitting ability (r = 0.661). However, the model for Y_2_ provides sufficient evidence of a linear dependence on the parameters X_2_, time of extraction, and X_3_, temperature, and no significant dependence on the interaction term X_2_ X_3_ accounting for the combined effect of extraction time and temperature.

Based on these results, we can consider the models for Y_1_ and Y_2_ validated. The difference between the predicted data and the experimental data is within the uncertainty limits associated with the used experimental procedure.

The results of our experiments showed that the proposed models could predict the trend of the expected extraction yield and (−)-(S)-UA recovery. According to our results, ethanol is the best solvent both for the extraction yield (Y_1_) and for UA recovery (Y_2_), while extraction time (X_2_) and temperature (X_3_) affect the responses to a lower extent. In particular, high temperature (80°C) and low extraction times (2 cycles x 5 min each) enhance (−)-(S)-UA recovery (Y_2_) and do not affect the extraction yield (Y_1_).

## 4. Conclusions

To sum up, in the present work, a suitable microwave-assisted extraction (MAE) coupled with HPLC-UV/PAD-CD protocol was set up for the stereochemical identification of UA content in the lichen extracts and to achieve a complete recovery of UA from *C. foliacea*. To the best of our knowledge, this is the first time microwaves have been exploited to extract UA. By combining analytical (HPLC), spectroscopical (mono- and bidimensional NMR), and chiroptical analysis (ECD and optical rotation value) and by comparison with the corresponding commercially available (R)-enantiomer used as standard, the UA produced by the *C. foliacea* was unambiguously identified as (S)-UA with an optical purity > 99%. Moreover, the herein proposed HPLC-UV/PAD protocol is suitable for the quantitative determination of UA in lichen extracts, and the association of an in-line CD allows us to obtain at the same time information about the stereochemistry of the UA, thus discriminating between the two enantiomers of UA that the different lichen species may produce.

Afterward, a versatile microwave-assisted extractive (MAE) protocol, assisted by a design of experiment (DoE), was optimized to recover (S)-UA from *C. foliacea* quantitatively. The best result in terms of UA extraction yield was obtained using ethanol and heating twice at 80°C under microwave irradiation for 5 min. The herein proposed procedure is simple, rapid, low cost, and applicable to evaluate the content of UA also in other lichen extracts. Of note, the optimized protocol requires less than 20 min in total to perform (−)-(S)-UA extraction and quantification. The optimized MAE protocol was assessed for the scale-up extraction of UA, and it represents a suitable procedure to produce (S)-UA for biological or pharmaceutical studies or commercial purposes.

## Figures and Tables

**Table 1:** Experimental plan and response data.

Exp#	Solvent	N° of Cycles × Minutes	T°C	Y_1_ Extraction Yield (%)	Y_2_ (−)-(*S*)-UA Yield ^1^
1	Acetone	2 × 5	60	4.16	12.86
2	Acetone	3 × 5	60	3.40	12.88
3	Acetone	2 × 5	80	3.60	13.07
4	Acetone	3 × 5	80	3.31	11.73
5	Ethyl Acetate	2 × 5	60	3.93	12.49
6	Ethyl Acetate	3 × 5	60	2.76	10.28
7	Ethyl Acetate	2 × 5	80	2.50	13.04
8	Ethyl Acetate	3 × 5	80	1.70	11.64
9	Ethanol	2 × 5	60	6.63	12.78
10	Ethanol	3 × 5	60	5.84	11.54
11	Ethanol	2 × 5	80	6.73	13.51
12	Ethanol	3 × 5	80	7.29	12.79
13 *	Ethanol	2 × 5	70	6.15 ± 0.01	13.2 ± 0.7
Predicted **	Ethanol	2 × 5	70	7 ± 2	13 ± 2

LEGEND: Exp#, experiment number in standard order; ^1^ (-)-(S)-UA yield is reported as mg of extracted metabolite per g of lichen. * exp13 has been repeated three times, and results are reported as a mean ± confidence interval at the 95% level of probability. ** Model’s predicted data was reported as a mean ± confidence interval at the 95% probability level.

## Data Availability

Not applicable.

## Supplementary Materials

The following are available online, Figure S1: Fumarprotocetraric acid ^1^H-NMR spectrum; Figure S2: Atranorin ^1^H-NMR spectrum; Figure S3: 1H-NMR (A), HSQC (B), 13C-DEPT (C) and 13C-NMR (D) of (−)-UA after MAE procedure.

## Sample Availability

Samples of the compounds ... are available from the authors.
