# Supplementary material for: Microwave-Assisted Extraction and HPLC-UV-CD Determination of (S)-usnic Acid in Cladonia foliacea
Source: Molecules. 2021 Jan 16;26(2):455. doi: 10.3390/molecules26020455 (PMC7830470; doi:10.3390/molecules26020455)
Supplement: Supplementary file 1 [file molecules-26-00455-s001.pdf]

SUPPLEMENTARY MATERIALS

S1. NMR spectra of extracted metabolites

Here below the ^{1}H-NMR spectra of fumarprotocetraric acid and atranorin are reported. Results are in accordance with those reported in literature (Figure S1 and 2).

Fumarprotocetraric acid: ^{1}H-NMR in DMSO-d6, δ (ppm): 10.57 (s, 1H, COH), 6.82 (s, 1H, CH), 6.62 (s, 1H, CHCH), 6.62 (s, 1H, CHCH), 5.28 (s, 2H, CH2), 2.46 (s, 3H, CH3), 2.42 (s, 3H, CH3).

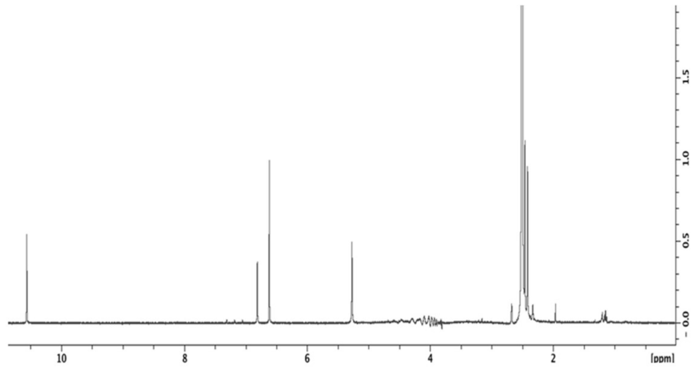

Figure S1: Fumarprotocetraric acid ^{1}H-NMR spectrum

Atranorin: ^{1}H-NMR in $CDCl_3$, δ (ppm): 12.59 (s, 1H, O**H**), 12.53 (s, 1H, O**H**), 11.98 (s, 1H, O**H**), 10.40 (s, 1H, CO**H**), 6.55 (s, 1H, C**H**), 6.44 (s, 1H, C**H)**, 4.02 (s, 3H, OC**H**$_3$), 2.72 (s, 3H, C**H**$_3$), 2.58 (s, 3H, C**H**$_3$), 2.13 (s, 3H, C**H**$_3$).

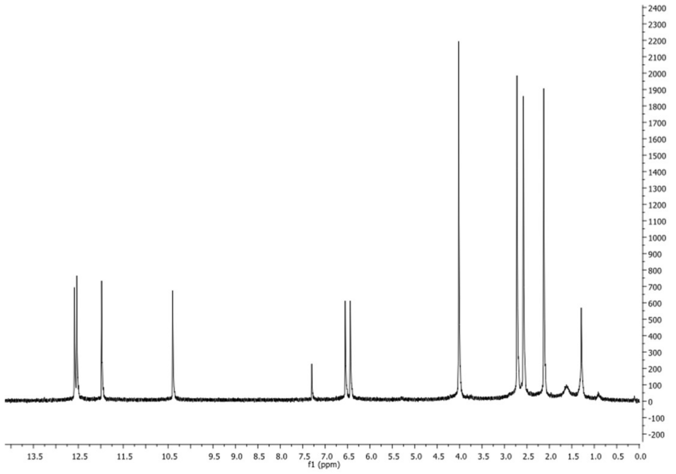

Figure S2: Atranorin ^{1}H-NMR spectrum

A full characterization of extracted (-)-(*S*)-usnic acid was performed via mono- and bi-dimentional NMR analysis. Results are coherent with those reported in literature (Figure S3).

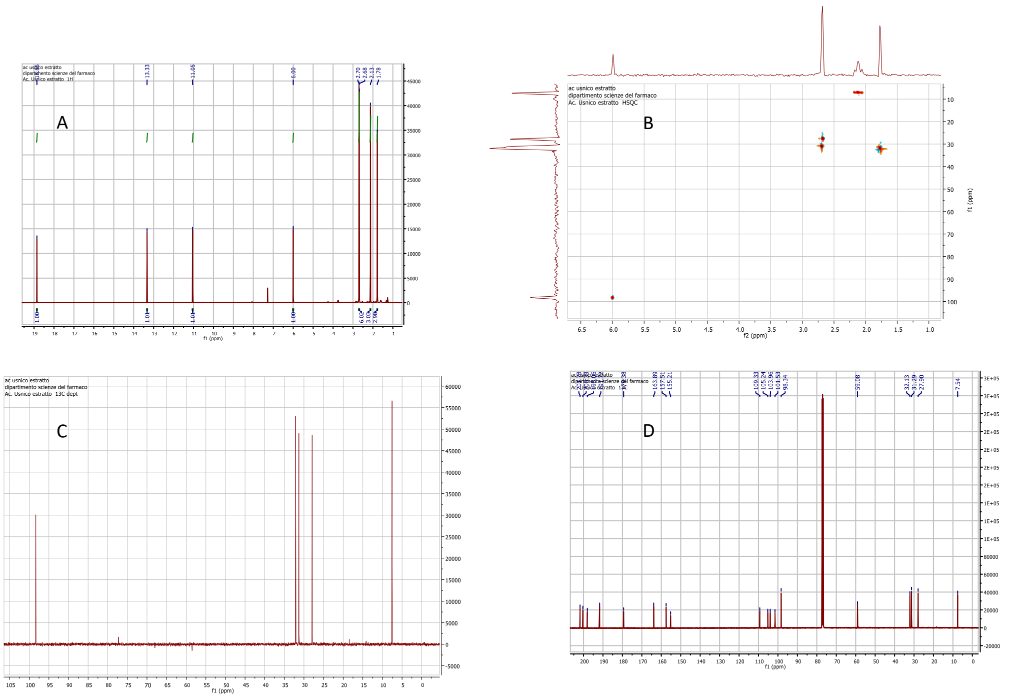

Figure S3: ^{1}H-NMR (A), HSQC (B), ^{13}C-DEPT (C) and ^{13}C-NMR (D) of (-)-usnic acid after MAE procedure
